# Iron Source Preference and Regulation of Iron Uptake in Cryptococcus neoformans

**DOI:** 10.1371/journal.ppat.0040045

**Published:** 2008-02-15

**Authors:** Won Hee Jung, Anita Sham, Tianshun Lian, Arvinder Singh, Daniel J Kosman, James W Kronstad

**Affiliations:** 1 The Michael Smith Laboratories, Department of Microbiology and Immunology, and Faculty of Land and Food Systems, University of British Columbia, Vancouver, British Columbia, Canada; 2 Department of Biochemistry, School of Medicine and Biomedical Sciences, The University at Buffalo, Buffalo, New York, United States of America; University of California San Francisco, United States of America

## Abstract

The level of available iron in the mammalian host is extremely low, and pathogenic microbes must compete with host proteins such as transferrin for iron. Iron regulation of gene expression, including genes encoding iron uptake functions and virulence factors, is critical for the pathogenesis of the fungus C*ryptococcus neoformans*. In this study, we characterized the roles of the *CFT1* and *CFT2* genes that encode C. neoformans orthologs of the Saccharomyces cerevisiae high-affinity iron permease *FTR1*. Deletion of *CFT1* reduced growth and iron uptake with ferric chloride and holo-transferrin as the *in vitro* iron sources, and the *cft1* mutant was attenuated for virulence in a mouse model of infection. A reduction in the fungal burden in the brains of mice infected with the *cft1* mutant was observed, thus suggesting a requirement for reductive iron acquisition during cryptococcal meningitis. *CFT2* played no apparent role in iron acquisition but did influence virulence. The expression of both *CFT1* and *CFT2* was influenced by cAMP-dependent protein kinase, and the iron-regulatory transcription factor Cir1 positively regulated *CFT1* and negatively regulated *CFT2*. Overall, these results indicate that C. neoformans utilizes iron sources within the host (e.g., holo-transferrin) that require Cft1 and a reductive iron uptake system.

## Author Summary

Opportunistic fungal pathogens and other invading microbes must overcome extreme iron limitation to proliferate in the mammalian host. It is not yet known which iron sources are preferred by fungal pathogens of mammals, although the mechanisms of acquisition are beginning to be explored. Some fungi produce iron-chelating siderophores to capture iron from host proteins, while others appear to require a membrane-bound iron permease–ferroxidase system. We describe the ability of the encapsulated yeast Cryptococcus neoformans to use host iron sources including transferrin and heme, and we identify an iron permease that is required for full disease progression in experimental mouse models. The permease is required for iron utilization from transferrin but not heme during growth in laboratory culture. This result when combined with the observed slow growth of the permease mutant during the experimental infections implicates transferrin as an important iron source in the host. However, we find that mutants lacking the permease eventually do cause disease, thus revealing that additional iron sources such as heme and other uptake mechanisms are available to C. neoformans. Finally, we noted that the permease mutant showed particularly poor growth in the brains of infected animals, suggesting that transferrin may be an especially important iron source in this tissue.

## Introduction

Pathogenic microbes such as the fungus Cryptococcus neoformans face a major challenge in acquiring iron during infection of vertebrate hosts. Free iron in tissues and fluids is maintained at extremely low levels due to the binding properties of the host proteins transferrin and lactoferrin. Moreover, vertebrates use iron deprivation as an important natural defense strategy against microbial pathogens [1]. For example, transferrin, which accounts for ∼1% of the total iron in the human body, is maintained at ∼33% saturation with iron in serum and effectively scavenges free iron [2]. Lactoferrin is similar to transferrin in structure and function but this protein retains iron in acidic conditions, such as at sites of inflammation, whereas transferrin binds iron at neutral pH [3]. Iron bound to heme is abundant in mammalian hosts but its availability during fungal pathogenesis is not yet clear because most of the heme is present in hemoglobin within erythrocytes. The data presented herein suggest that heme and transferrin may both be important iron sources for C. neoformans because each can support the growth of the fungus in culture.

The mechanisms by which microbes acquire iron during infection are of considerable interest and are best characterized in bacterial pathogens [4]. For example, many pathogenic bacteria produce siderophores that bind to ferric iron with high affinity, and many are able to utilize ferritin, transferrin, lactoferrin, heme and heme-containing proteins. In many species, the mechanisms of iron acquisition have been elucidated in detail and preferences for specific iron sources during infection are being identified. For example, Staphylococcus aureus preferentially uses iron from heme rather than from transferrin during infection [5].

Mechanisms of iron acquisition are less well studied in pathogenic fungi. However, iron transport pathways have been well characterized in the model fungus Saccharomyces cerevisiae, which has at least two distinct high-affinity uptake systems. One is a reductive pathway in which ferric iron is reduced to ferrous iron by cell surface reductase activity with subsequent transport across the plasma membrane by the high-affinity iron permease (Ftr1)–multicopper ferroxidase (Fet3) complex [6–8]. The second high-affinity iron transport pathway uses siderophores from other organisms and transports iron bound to these molecules via cell surface transporters encoded by the *ARN* gene family [9–11].

Similar iron transport pathways exist in fungal pathogens of humans, although these have been less well studied. For example, both the reductive and siderophore iron uptake systems are found in Candida albicans and Aspergillus fumigatus. Two orthologous genes of the high-affinity iron permease *FTR1* were identified in C. albicans and one of them, *CaFTR1*, was shown to be required for systemic infection [12]. *CaFTR1* also mediates iron acquisition from transferrin [13]. C. albicans is not able to synthesize siderophores but has siderophore transporters such as CaArn1/CaSit1. However, the role of CaArn1/CaSit1 in an infected host may be minimal because a mutant only showed defects in epithelial invasion [14]. C. albicans does have hemolytic activity and utilizes heme and hemoglobin as iron sources [15–17]. Furthermore, cell surface proteins that bind heme and hemoglobin have been identified in C. albicans [18]. Additionally, this yeast uses hemoglobin as a signaling molecule to alter gene expression and to induce adhesion to host cells, and also to trigger the yeast to hyphae transition that is required for pathogenesis [19]. However, the question of whether heme or hemoglobin utilization plays a role in virulence *in vivo* is still unclear because none of previous studies showed virulence effects. Another animal pathogen, A. fumigatus also possesses the high-affinity iron permease FtrA, but this enzyme is not required for virulence. In contrast, the A. fumigatus SidA protein, which is responsible for siderophore synthesis, is essential for virulence [20,21].

Regulatory mechanisms that govern expression of the high-affinity iron permease have also been investigated. In S. cerevisiae, the global transcriptional activator Aft1 activates expression of *FTR1* and other genes of the iron regulon [22,23]. Interestingly, orthologs of *S. cerevisiae FTR1* in other fungi are negatively regulated by a conserved GATA-type zinc finger protein. Thus, the iron permease gene *FIP1* is regulated by Fep1 in Schizosaccharomyces pombe and the permease gene *FER2* is regulated by Urbs1 in Ustilago maydis [24,25]. We recently identified and characterized a global transcriptional regulator, Cir1, in C. neoformans that shows sequence and functional similarities to Fep1 and Urbs1. We found that Cir1 regulates many genes for iron acquisition including genes for putative high-affinity iron permeases, as well as genes involved in virulence in C. neoformans [26].

It has been suggested that the cAMP pathway influences iron uptake by controlling expression of the high-affinity iron permease in fungi. In S. cerevisiae, the catalytic subunit of cAMP-dependent protein kinase (PKA), Tpk2, negatively regulates expression of *FTR1* and *FET3*, and Tpk2 may indirectly control respiratory growth by negative regulation of iron uptake [27]. This connection between respiration and iron uptake is supported by the finding that the *aft1* mutant fails to grow in the presence of non-fermentable carbon sources [28,29]. A connection between cAMP and iron uptake also exists in U. maydis because the expression of *FER2* is positively regulated by the cAMP pathway [24]. Similar regulatory connections exist in C. neoformans because transcriptome studies demonstrated that genes for reductive iron uptake are differentially expressed in mutants lacking components of the cAMP pathway [30].

*C*. *neoformans* utilizes several transport systems to acquire iron from the environment and both high and low affinity iron uptake activities mediated by cell surface reductases have been detected [31]. Nonenzymatic reduction of ferric iron by a secreted reductant, 3-hydroxyanthranilic acid, and by melanin in the cell wall, may also contribute to iron acquisition [32]. C. neoformans reportedly does not produce siderophores but is capable of utilizing iron bound to siderophores secreted from other microorganisms [31]. The *SIT1* gene, which encodes a siderophore transporter, was found to mediate siderophore utilization, but the gene is not required for virulence [33].

In this study, we identified and functionally characterized two candidate iron transporters in C. neoformans, *CFT1* and *CFT2 (Cryptococcus* Fe Transporter), which are orthologs of *S. cerevisiae FTR1*. Mutants lacking *CFT1* and/or *CFT2* were constructed and characterized for their ability to use host iron sources and to cause disease. We found that *CFT1* is involved in a reductive iron uptake pathway that is required for utilization of transferrin. *CFT1* is also required for full virulence thus indicating that C. neoformans may preferentially utilize transferrin in a tissue specific manner, especially in the brain. *CFT2* does not appear to play a role in iron acquisition under the conditions we tested but did contribute to virulence. We also demonstrate that *CFT1* and *CFT2* are differentially regulated by Cir1 and that transcript levels of both genes are influenced by the cAMP pathway.

## Results

### C. neoformans Possesses Two Orthologs of the S. cerevisiae High-Affinity Iron Permease Ftr1

We initially searched the genome of the highly virulent strain H99 (serotype A) of C. neoformans to identify orthologs of the S. cerevisiae high-affinity iron permease Ftr1 [8,34]. Two highly conserved paralogous candidate genes were identified and the gene on chromosome 12 was designated *CFT1* (*Cryptococcus* Fe Transporter 1) while the gene on chromosome 3 was named *CFT2*. We had previously identified *CFT1* (but not *CFT2*) as a candidate iron permease gene in transcriptional profiling experiments using serial analysis of gene expression (SAGE) and microarrays to examine the response to iron levels, cAMP signaling and experimental meningitis [30,35,36]. In the study of Lian et al., (2005), disruption of *CFT1* in a serotype D strain background yielded a mutant with poor growth on low iron medium. Interestingly, *CFT2* was also found among genes with induced expression upon phagocytosis [37].

**Figure 1:**
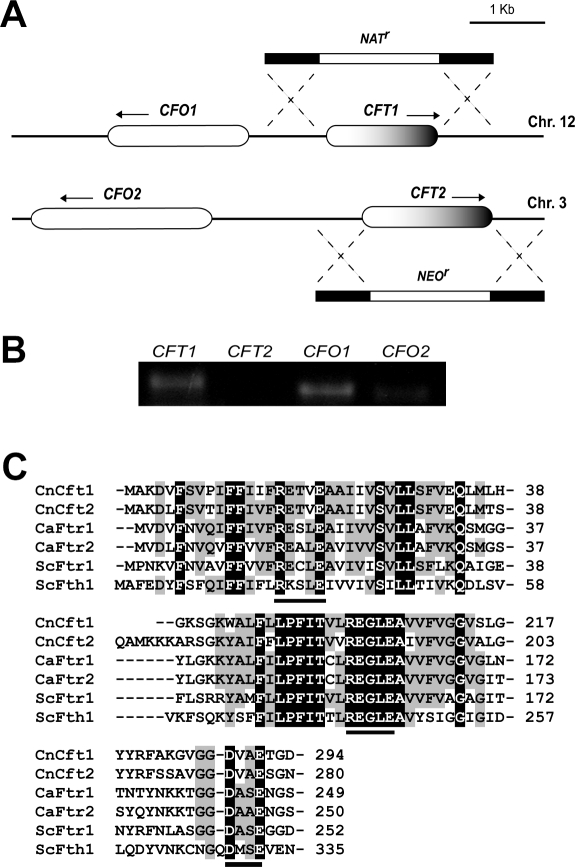
Genomic Arrangement and Conserved Regions of *CFT1* and *CFT2* (A) *CFT1* and *CFT2* are located on chromosomes 12 and 3, respectively. The genes were disrupted by transforming the disruption cassette containing the selectable marker for resistance to nourseothricin (*NAT* ^r^) or neomycin (*NEO* ^r^) as shown. (B) Basal levels of the transcripts are shown for the genes indicated above the lanes. The cells were grown under low-iron conditions and transcripts were amplified by RT-PCR (30 cycles). (C) Comparison of specific regions of the predicted amino acid sequences of Cft1 and Cft2 with the high-affinity iron permeases in other fungi. Both Cft1 and Cft2 contain the highly conserved motifs underlined. CnCft1: C. neoformans Cft1 from strain H99; CnCft2: C. neoformans Cft2 from strain H99; CaFtr1: C. albicans CaFtr1 (AAF69680); CaFtr2: C. albicans CaFtr2 (AAF69681); ScFtr1: S. cerevisiae Ftr1 (NP_011072); ScFth1: S. cerevisiae Fth1 (CAA85171).

A comparison of the predicted amino acid sequences showed 36% identity and 54% similarity between Ftr1 of S. cerevisiae and Cft1, 36% identity and 53% similarity between Ftr1 and Cft2, and 53% identity and 66% similarity between Cft1 and Cft2. Similarities in genome arrangements exist for *CFT1* and *CFT2* in that *CFT1* was paired with an adjacent putative ferroxidase gene designated *CFO1* (*Cryptococcus* Ferroxidase 1) and *CFT2* was paired with the putative ferroxidase gene *CFO2*. Both ferroxidases showed high similarity to the Fet3 protein of S. cerevisiae (data not shown). *CFT1* and *CFO1* are transcribed bi-directionally as are *CFT2* and *CFO2,* with 791 bp and 2103 bp promoter regions, respectively (Figure 1A). Measurements of basal transcript levels of the genes under low-iron conditions (by RT-PCR) showed that the transcripts of *CFT1* and *CFO1* were readily detected whereas *CFO2* was less abundant and *CFT2* was undetectable (Figure 1B). We focused on the roles and regulation of *CFT1* and *CFT2* for this study.

Severance *et al*. (2004) showed that S. cerevisiae Ftr1 has seven transmembrane domains with an orientation of N-terminal outside and C-terminal inside the cell [38]. Furthermore, their study suggested that two motifs, REXLE and DASE, are essential for iron transport and are strongly conserved among other fungal Ftr1 homologs. In this context, we analyzed the transmembrane (TM) helix number and TM topology of Cft1 and Cft2 in silico with the protein localization prediction program Localizome and found that both proteins have seven predicted TM domains and the same predicted topology as Ftr1 in S. cerevisiae [39]. Amino acid alignments with the S. cerevisiae proteins Ftr1 and the vacuolar iron transporter Fth1, and also with the C. albicans Ftr1 and Ftr2 proteins, showed that both Cft1 and Cft2 possess the highly conserved motifs thought to be essential for iron transport (Figure 1C).

### Expression of *CFT1* and *CFT2* Is Iron Dependent and Is Oppositely Regulated by Cir1

**Figure 2:**
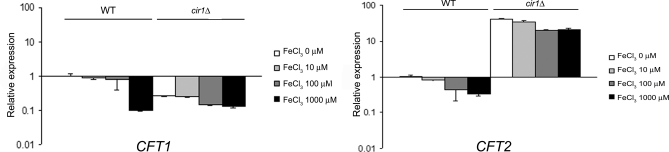
Expression of *CFT1* and *CFT2* Is Iron Dependent and Differentially Regulated by Cir1 Transcriptional regulation of *CFT1* and *CFT2* in the wild-type strain and the *cir1* mutant was monitored by quantitative real-time RT-PCR after growing cells in medium containing various concentrations of iron (0, 10, 100 and 1,000 μM of FeCl_3_). Data were normalized by using *ACT1* as an internal control and are presented as relative expression. Data are from four replicates and bars represent the standard deviations.

Transcript levels of the high-affinity iron permease genes are influenced by iron levels in S. cerevisiae, C. albicans and S. pombe [12,22,25]. Therefore, we tested whether *CFT1* and *CFT2* are regulated in a similar manner in C. neoformans. Wild-type cells were cultured in different concentrations of iron (0, 10, 100 and 1000 μM), and expression of *CFT1* or *CFT2* was measured by real-time RT-PCR. Transcript levels of both *CFT1* and *CFT2* were reduced as iron levels increased in cultures of the wild-type strain (Figure 2). Therefore, it appears that C. neoformans responds to iron deprivation by increasing transcription of these candidate iron permease genes. Note that although the transcript levels for *CFT2* were influenced by iron, the significance for *CFT2* function is unclear because the basal transcriptional level of *CFT2* in low iron media was 100-fold lower than that of *CFT1* (see also Figure 1B). It may be that Cft2 has a minor or redundant function in iron uptake relative to Cft1, that the gene plays a role in other growth conditions, or that Cft2 functions to transport iron from stores in the vacuole*.* Additionally, *CFT1* and *CFT2* transcript levels were reduced 10-fold and 3-fold respectively in the wild-type strain during growth in 1000 μM of iron, and this regulatory response may also suggest a minor or different role for *CFT2* in iron uptake.

We previously showed that iron permease genes are downstream targets of the iron regulatory transcription factor Cir1 in a C. neoformans serotype D strain by microarray analysis. Northern analysis also suggested that *CFT2* transcript levels were influenced by Cir1 in a serotype A strain [26]. To further examine the regulation by Cir1, we performed transcriptional analysis to determine whether both *CFT1* and *CFT2* are downstream of Cir1 in the serotype A strain. Transcription of *CFT1* and *CFT2* was monitored by real-time RT-PCR in the *cir1* mutant grown in media with different concentrations of iron. Our results showed that *CFT1* transcript levels were reduced in the *cir1* mutant, indicating positive regulation by Cir1 (Figure 2). Furthermore, the *CFT2* transcript was higher in the *cir1* mutant indicating negative regulation by Cir1 (Figure 2) and this result is consistent with our previous observations [26]. In addition, it appears that when Cir1 is deleted, the transcript levels of *CFT1* and *CFT2* are no longer responsive to iron concentrations compared with the wild-type strain.

### *CFT1* Is Necessary for Growth on Iron Sources that Require Reductive Uptake

To characterize the functions of *CFT1* and *CFT2*, we generated mutants lacking each of the genes. Double mutants lacking both genes were also constructed to potentially unmask phenotypes hidden by redundancy (see Materials and Methods). Reconstituted strains with a reintroduced wild-type copy of *CFT1* or *CFT2* at the original locus were also constructed and analyzed. Initial tests indicated that several independently generated *cft1* and *cft2* mutants showed similar growth rates in YPD medium at 30°C. These tests also revealed that the mutants did not differ from wild type with regard to capsule formation in low iron medium, melanin synthesis and growth at 37°C. The *cft1 cft2* double mutants also did not display changes in capsule or melanin production, but did display a reduced growth rate in YPD medium (Figure S1).

**Figure 3:**
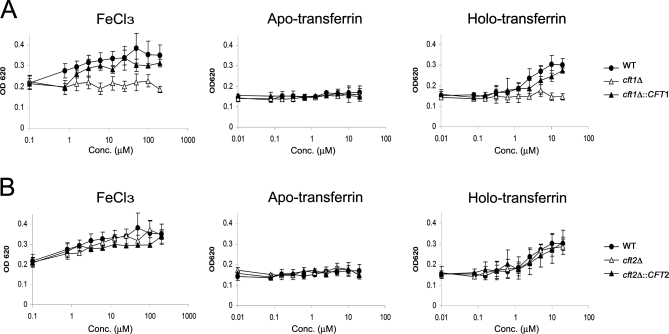
*CFT1* Is Required for Reductive Iron Uptake (A) Iron-starved strains (the wild-type, the *cft1* mutant and the *CFT1* reconstituted strain) were inoculated into media containing FeCl_3_, apo-transferrin or holo-transferrin, which were added in stepwise 2-fold dilutions. The actual range of concentrations in the cultures was between 200 μM and 0.78 μM of FeCl3, and 20 μM and 0.078 μM of apo- or holo-transferrin. The OD620 reading at 0.1 μM in FeCl3 containing plates and 0.01 μM in Apo- or Holo-transferrin containing plates represent the level of growth in media without an added iron source, starting at the standard inoculum density of 0.08. All cultures were incubated at 30°C and turbidity was measured after 72 h. The averages of three independent experiments are presented with bars representing the standard deviations. (B) The same experiments as shown in (A) were performed for the *cft2* mutant and all strains showed similar patterns of growth.

**Figure 4:**
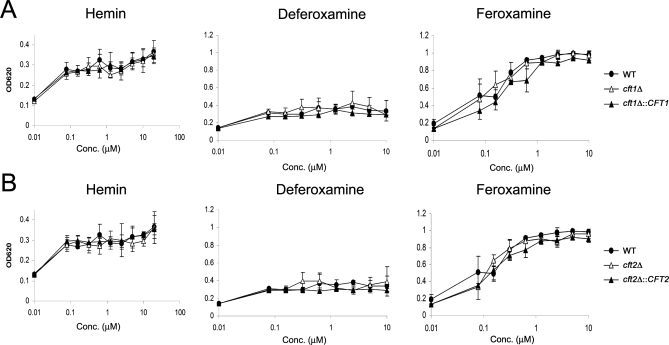
Non-Reductive Iron Uptake Systems Are Independent of Cft1 and Cft2 (A) Iron-starved strains (the wild-type, the *cft1* mutant and the *CFT1* reconstituted strain) were inoculated into media containing heme, deferoxamine and feroxamine, which were added in stepwise 2-fold dilutions. The range of concentrations was between 20 μM and 0.78 μM (heme) and between 10 μM and 0.78 μM (deferoxamine and feroxamine); note that the scale on the X-axis is between 0.01 and 10 or 100 μM. In each graph, the OD620 reading at 0.01 μM indicates the level of growth in media without an added iron source starting at the standard inoculum density of 0.08. All cultures were incubated at 30°C and turbidity was measured after 72 h. Averages of three independent experiments are presented with bars representing the standard deviations. (B) The same experiments as shown in (A) were performed for the *cft2* mutant and all strains showed similar patterns of growth.

The wild-type strain, each single mutant and the reconstituted strains were tested for utilization of different iron sources in vitro. Strains were first grown in low-iron medium to reduce intracellular iron stores, and then were transferred to fresh low-iron medium and low-iron medium supplemented with the inorganic iron salt FeCl_3_, apo-transferrin, holo-transferrin, heme or siderophores. Transferrin and heme were of particular interest because of their abundance as iron sources in mammals. The different concentrations of iron sources were prepared by serial dilution to ensure that any growth phenotypes observed could be correlated to a dependence on the iron source. The wild-type strain grew well with all iron sources and showed particularly robust growth in the presence of the iron-loaded siderophore, feroxamine (Figures 3 and 4). As expected, little or no growth was observed with apo-transferrin and the siderophore deferoxamine that lacks iron. The *cft2* mutants behaved like the wild-type strain in all conditions (Figures 3B and 4B). In contrast, the *cft1* mutants showed reduced growth in the presence of inorganic iron (FeCl_3_) or holo-transferrin, but not heme or feroxamine (Figures 3A and 4A). Moreover, the *cft1* mutant displayed growth defects for all concentrations of FeCl3 or holo-transferrin, a result consistent with the idea that Cft1 may be a high-affinity iron permease in C. neoformans. An analysis of the time course of growth for the strains also confirmed the growth defect of the *cft1* mutant with FeCl_3_ as the iron source, and demonstrated that the *cft1 cft2* double mutant behaved like the *cft1* mutant in this assay (Figure S2A). These findings suggest that *CFT1* is required for the reductive iron uptake pathway in C. neoformans because ferric iron and iron from transferrin are believed to be transported via this pathway. Given that transferrin is a major iron carrier in the mammalian host, *CFT1* may play a key role for iron acquisition during infection. These results also indicate that uptake of siderophore-bound iron is independent of *CFT1* and *CFT2*.

### A *cft1* Mutant Is Attenuated for Iron Uptake from FeCl_3_ and Transferrin

**Figure 5:**
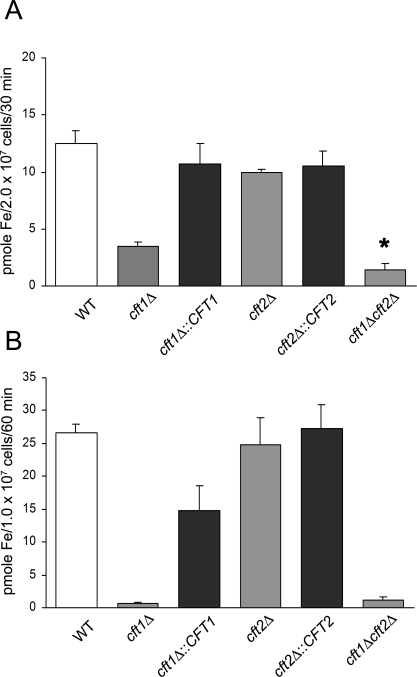
Iron Uptake Is Impaired in the *cft1* Mutant (A) Strains were grown in defined low-iron medium and analyzed for iron uptake with ^55^FeCl_3_. The results show the average from three experiments with bars representing the standard deviations. The asterisk (*) indicates that the iron uptake for the *cft1 cft2* double mutant was statistically different from that of the *cft1* mutants (*p* = 0.007 by a Student *t* test). (B) The strains indicated were grown in low-iron medium and uptake of ^55^Fe from transferrin was measured. The results shown are an average from three experiments with bars representing the standard deviations.

The ability of the wild-type and mutant strains to take up iron was directly compared by assaying accumulation of iron from ^55^FeCl_3_ and from ^55^Fe-loaded transferrin (Figure 5). In the assay with ^55^FeCl_3_, iron uptake by the *cft1* mutant and the *cft1 cft2* double mutant occurred at only 27% and 11% of the level found for the wild-type strain, respectively, suggesting that Cft1 plays a primary role in iron uptake in C. neoformans (Figure 5A). Although Cft2 appeared to make a contribution based on the lower uptake of the double mutant, the *cft2* mutant did not show a statistically significant reduction in uptake compared to the wild-type strain. Cft1 also played a major role in the acquisition of ^55^Fe from transferrin because negligible uptake was detected compared with the wild-type or reconstituted strains (Figure 5B). The *cft1 cft2* double mutant again behaved like the *cft1* mutant and no influence on uptake was seen in the strain lacking *CFT2*. Overall, these results highlight the role of Cft1 in iron uptake and are consistent with the poor growth of the *cft1* mutant on iron sources that require reductive uptake.

### Loss of *CFT1* Influences Transcript Levels for *CFT2* and the Siderophore Transporter Gene *SIT1*

Loss of the high affinity iron uptake system would be expected to cause lower intracellular iron availability and to potentially influence the expression of iron-responsive genes. In addition, studies in S. cerevisiae indicate that modifying the expression of Fet4, which is responsible for low-affinity iron uptake, can modulate expression of components of the high-affinity iron uptake system. Specifically, disruption of *FET4* increases the activity of the high affinity uptake system and overexpression of *FET4* decreases the activity [40]. In light of these observations, we investigated transcript levels for *CFT1* in the *cft2* mutant and for *CFT2* in the *cft1* mutant. We also analyzed levels of the *SIT1* transcript in the *cft1* and *cft2* mutants to determine whether alteration of the reductive iron uptake pathway influenced the non-reductive siderophore uptake pathway.

**Figure 6:**
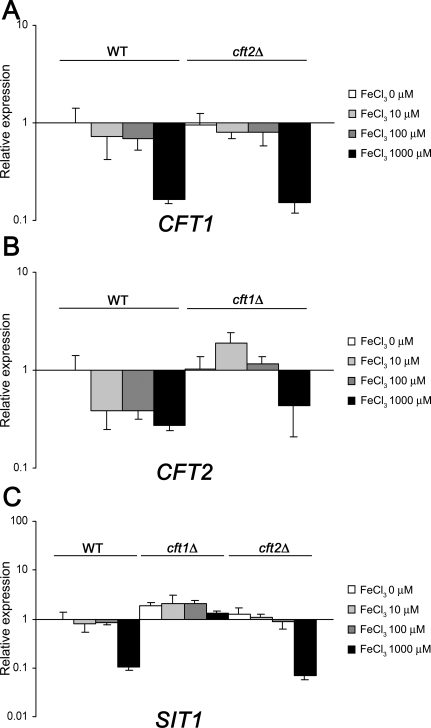
*CFT1* Influences Transcript Levels for *CFT2* and the Siderophore Transporter Gene *SIT1* (A) Transcriptional regulation of *CFT1* in the *cft2* mutant was monitored by quantitative real-time RT-PCR after growing cells in media containing different concentrations of iron (0, 10, 100 and 1,000 μM of FeCl_3_). (B) Transcriptional regulation of *CFT2* in the *cft1* mutant was monitored by quantitative real-time RT-PCR in the same culture conditions. (C) Transcription of *SIT1* was analyzed in both the *cft1* and the *cft2* mutant strains in parallel experiments. All data were normalized by using *ACT1* as an internal control, according to the ΔΔCt method, and are presented as fold changes (*y*-axis). Data are from four replicates and bars represent the standard deviations.

The wild-type strain and the *cft1* and *cft2* mutants were grown in media containing different concentrations of iron and the transcript levels of *CFT1*, *CFT2* and *SIT1* were measured by real-time RT-PCR. We found that the transcript levels of *CFT1* displayed similar and expected expression patterns in both the wild-type strain and the *cft2* mutant; specifically, mRNA levels of *CFT1* decreased as the iron concentration increased (Figure 6A). In contrast, *CFT2* transcript levels were elevated in the *cft1* mutant, especially in the presence of 10 μM and 100 μM iron, compared to the reduced levels seen in the wild-type strain in response to iron (Figure 6B). This result supports the conclusion that loss of Cft1 leads to reduced intracellular iron levels. It is possible that the elevated *CFT2* transcript levels resulting from loss of *CFT1* could potentially result in more Cft2 product and influence the iron acquisition capabilities of the cells.

For the *SIT1* gene, both the wild-type strain and the *cft2* mutant showed an iron-dependent reduction of *SIT1* transcript levels (Figure 6C). However, *SIT1* levels were higher in the *cft1* mutant compared to other strains, and the levels were no longer entirely responsive to environmental iron concentration. These results again imply that the *cft1* mutant is debilitated in its ability to import iron and that intracellular iron levels may become constitutively low. This situation would potentially account for the unresponsive expression of *SIT1* even in the presence of 1000 μM of iron. This is in contrast to the decrease in the *CFT2* transcript at the same iron concentration and this difference may reflect distinct mechanisms of iron regulation for the two genes. As mentioned above, *CFT2* is negatively regulated by the iron-responsive transcription factor Cir1 and we have shown previously that *SIT1* is positively regulated [26]. Overall, our results suggest that disruption of *CFT1* influences expression of *CFT2* and *SIT1*, a result that is consistent with a reduction in intracellular iron levels in the *cft1* mutant.

### Regulation of *CFT1* and *CFT2* by PKA

**Figure 7:**
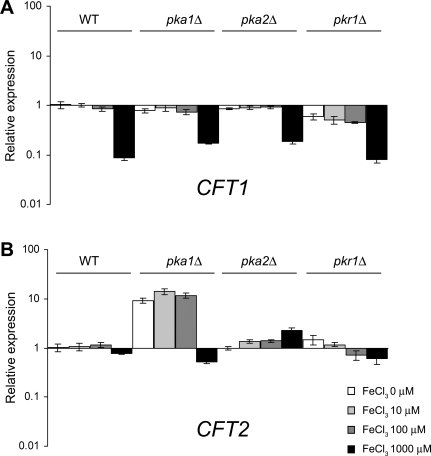
The Expression of *CFT1* and *CFT2* Are Regulated by Components of the cAMP-Dependent Protein Kinase Strains lacking *PKA1*, *PKA2* or *PKR1* were grown in media containing different concentrations of iron (0, 10, 100 and 1,000 μM of FeCl_3_), and transcript levels of *CFT1* (A) or *CFT2* (B) were compared by quantitative real-time RT-PCR. Data were normalized by using *ACT1* as an internal control, according to the ΔΔCt method, and presented as relative expression. Data are from four replicates and bars represent the standard deviations.

The cAMP pathway controls the expression of high-affinity iron permeases in S. cerevisiae and U. maydis. However, these two fungi display opposite regulatory patterns. In S. cerevisiae, expression of the gene for the high-affinity iron permease, *FTR1,* is negatively regulated whereas in U. maydis, expression of the orthologous gene, *FER2,* is positively regulated [24,27]. These observations led us to investigate regulatory mechanisms of the high-affinity iron permease genes *CFT1* and *CFT2* in relation to the cAMP pathway in C. neoformans. For this analysis, strains lacking the genes encoding the catalytic subunits (*PKA1*, *PKA2*) or the regulatory subunit (*PKR1*) of PKA were grown in media containing different concentrations of iron, and transcript levels of *CFT1* and *CFT2* were compared by real-time RT-PCR. The results revealed a similar expression pattern for *CFT1* in the wild-type strain and the *pka1* and *pka2* mutants such that the transcript level was dependent on iron concentration. However, *CFT1* expression was found to be reduced by 2- to 3-fold in the *pkr1* mutant compared to the wild-type strain (Figure 7A). Conversely, the transcript levels of *CFT2* were found to be elevated upon deletion of *PKA1* (Figure 7B). These results indicate that the cAMP pathway negatively regulates the transcript level of *CFT2* via Pka1 in C. neoformans and suggest that PKA also negatively influences *CFT1* expression but to a lesser extent. As mentioned earlier, the basal transcript level for *CFT2* is much lower than that of *CFT1* and this may influence both the detection and the biological relevance of PKA regulation of these genes. It is interesting, however, that the loss of different subunits of PKA influences transcript levels for the two genes, and that reciprocal patterns of regulation for loss of the catalytic and regulatory subunits were not observed. These results suggest that the influence of the cAMP pathway may be exerted by different mechanisms for each of the genes.

### The *cft1* Mutant Has Increased Susceptibility to Inhibitors of Fungal Sterol Metabolism

**Figure 8:**
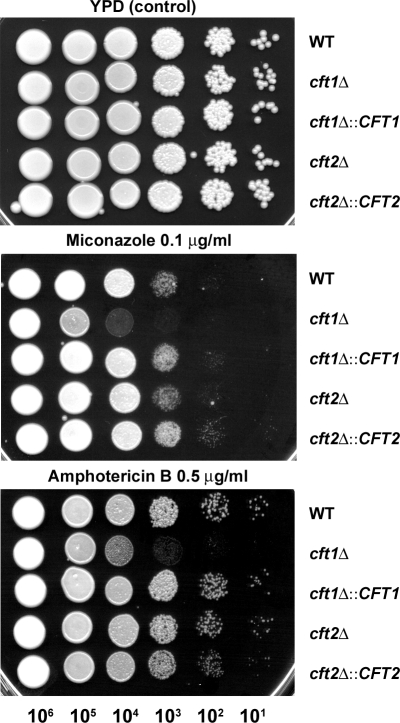
Deletion of *CFT1* Increases Susceptibility to Antifungal Agents that Target Fungal Sterol Biosynthesis and Function The growth of strains in media containing the antifungal drugs miconazole and amphotericin B was monitored to assess sensitivity. Ten-fold serial dilutions of cells (starting at 10^6^ cells) were spotted onto YPD plates with and without the antifungal drug indicated. Plates were incubated at 30°C for two days.

A relationship between iron levels and susceptibility to antifungal drugs that act at the level of ergosterol biosynthesis has been reported [41]. Specifically, the availability of iron influences susceptibility of C. albicans to antifungal drugs, and strains lacking the iron permease CaFtr1 display increased sensitivity to fluconazole due to alteration of expression of genes in the ergosterol synthesis pathway [41]. A similar connection also exists in S. cerevisiae because cytosolic iron levels influence C-4 methyl sterol oxidase (Erg25), an enzyme that is essential for ergosterol synthesis [42]. In this context, we tested the C. neoformans iron permease mutants to investigate whether a deficiency in iron uptake alters susceptibility to antifungal drugs. The *cft1* mutant displayed increased sensitivity to the azole, miconazole, suggesting that a deficiency in iron uptake may also influence ergosterol synthesis in C. neoformans (Figure 8). This phenotype is also supported by the observation that *cft1* mutants showed an increase in sensitivity to the antifungal drug amphotericin B, compared to the wild-type strain (Figure 8). These findings suggest that inhibitors of Cft1 activity could provide a synergistic effect when used in combination with existing antifungal drugs, and that Cft1 could be a novel drug target for treatment of cryptococcosis.

### The *cft1* Mutant Is Attenuated for Virulence and for Colonization of the Brain

**Figure 9:**
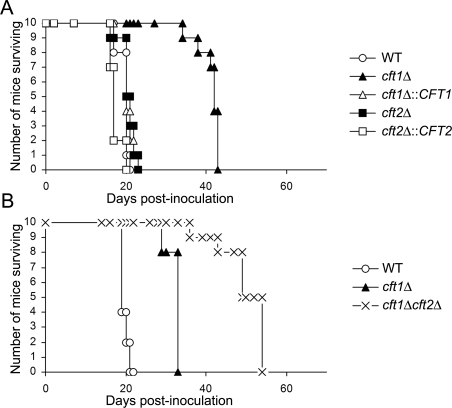
The *cft1* and *cft1 cft2* Mutants Are Attenuated for Virulence (A) Ten female A/Jcr mice were infected intranasally with each of the strains indicated and the survival of the mice was monitored over the time course indicated on the *x*-axis. The difference in survival between the *cft1*Δ mutant and the WT strain was significant based on a Kaplan-Meier survival analysis (*p* < 0.0001). (B) In a separate experiment, the virulence of the *cft1*Δ *cft2*Δ double mutant was compared with that of the WT strain and the *cft1*Δ mutant, again with 10 mice per inoculation. A second, independently isolated, double mutant showed a similar attenuation of virulence. All of the virulence tests were performed at least twice. The difference in survival between the *cft1*Δ mutant and the WT strain was significant (*p* < 0.0001), as was the difference between the *cft1*Δ mutant and the *cft1*Δ *cft2*Δ double mutant (*p* < 0.0001).

The deficiency of the *cft1* mutant in growth and iron acquisition with transferrin prompted an examination of the virulence of the *cft1* and *cft2* mutants in the mouse inhalation model of cryptococcosis. We found that mice infected with the wild-type strain, the *cft2* mutant, and the *CFT1* or the *CFT2* reconstituted strains, showed 100% mortality by ∼20 days. However, mice infected with the *cft1* mutant survived to ∼43 days indicating that deletion of *CFT1* caused a significant attenuation of virulence (Figure 9A). These results support the conclusion that Cft1 plays a role in iron acquisition in vivo. Furthermore, because the *cft1* mutants showed defects in transferrin but not in heme and siderophore utilization *in vitro*, we hypothesize that transferrin is an important iron source for C. neoformans during growth in mammalian hosts. The virulence attributes of the *cft1* single and the *cft1 cft2* double mutants were also compared in a separate experiment. We found that mice infected with the double mutant survived to ∼54 days, a time significantly longer than mice infected with the *cft1* single mutants (Figure 9B). These results suggest that Cft2 makes a contribution during infection that is evident in the absence of *CFT1*.

**Figure 10:**
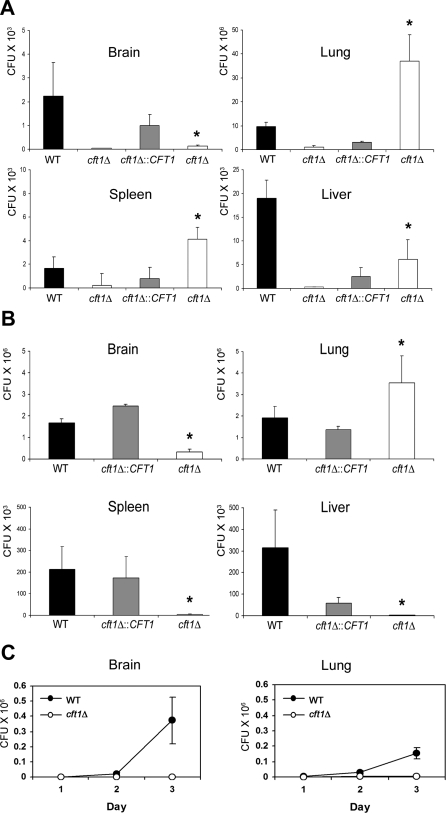
The *cft1* Mutant Shows Reduced Tissue Colonization in a Mouse Model of Cryptococcosis (A) Distribution of fungal cells in the organs of mice infected by the inhalation method. Organs from mice infected with the wild-type strain, the *cft1* mutant or the *CFT1* reconstituted strain were collected at day 19 when the wild type and the reconstituted strain reached the end point. Additionally, organs from mice infected with the *cft1* mutants were collected at day 34 when mice reached the end point. Fungal burdens were monitored in organs by determining colony-forming units (CFU) upon plating on YPD medium. Three mice for each strain were used at each time point. Data are average CFUs per organ with standard deviations. CFUs for the *cft1* mutant at day 34 are marked with an asterisk; the other counts are from day 19. (B) Distribution of fungal cells in mice infected by the tail vein injection method. Organs from mice infected with the wild-type strain or the *CFT1* reconstituted strain were collected at day 7 when the wild type and the reconstituted strain reached the end point. Organs from mice infected with the *cft1* mutants were collected at day 29 when mice reached the end point. Fungal burdens were monitored as described in (A). Three mice for each strain were used. Data are averages with standard deviations. CFUs for the *cft1* mutants at day 29 are marked with an asterisk; the other counts are from day 7. (C) The ability of the *cft1* mutant to colonize brain and lung tissue was compared with the wild-type strain. Mice were inoculated with 2.5 × 10^5^ cells by tail vein injection, sacrificed at the times indicated and analyzed for fungal burden.

The distribution of fungal cells in the infected mice was also assessed to determine the ability of the *cft1* mutant to colonize host tissue. Organs from mice infected with the wild-type strain, the *cft1* mutant or the *CFT1* reconstituted strain were collected and fungal burden was measured by determining colony-forming units (CFU). We harvested the organs at days 19 and 34 because mice infected with the wild-type and reconstituted strains succumbed to infection on or around day 19 and mice infected with the *cft1* mutant started to reach the endpoint at approximately day 34. These time points for the inhalation model of cryptococcosis would therefore represent the maximum disease progression in terms of fungal dissemination and proliferation. As shown in Figure 10A, wild-type and reconstituted cells were widely disseminated throughout the host at day 19. However, in mice infected with the *cft1* mutant, the number of the fungal cells was much lower at day 19 suggesting a defect in dissemination and/or colonization of organs (Figure 10A). We hypothesize that the failure of the *cft1* mutant to disseminate or colonize during infection is due, at least in part, to an inability to use transferrin. The *cft1* mutant eventually disseminated from the lung to the spleen and the liver in infected mice by day 34 (Figure 10A), implying that the *cft1* mutants can presumably utilize host iron sources other than transferrin (e.g., heme). In particular, the fungal burdens for the *cft1* mutant were higher at day 34 in the lung, spleen and liver (30-, 17- and 21-fold, respectively) compared to day 19. However, the number of fungal cells in the brains of mice infected with the *cft1* mutant remained low and was only two-fold higher at day 34 compared to day 19 (Figure 10A). This observation implies that transferrin or similar iron sources transported by *CFT1* via the reductive iron uptake pathway may be primary iron sources for C. neoformans in the brain. It is likely that the *cft1* and the *cft1 cft2* mutants can use other iron sources in infected mice, and heme is a likely candidate because these mutants grow well with heme as the sole iron source in vitro (Figures 4A and S2).

We also tested whether a different route of inoculation of fungal cells by tail vein injection could influence the behavior of the *cft1* mutant in vivo. Mice infected with the wild-type and the *CFT1* reconstituted strains succumbed within 7 days. In contrast, mice infected with the *cft1* mutant survived to day ∼29 (data not shown) thus revealing that the *cft1* mutant was also attenuated for virulence in this model of cryptococcosis. In general, approximately three orders of magnitude more fungal cells were found in the brains of the mice infected via tail vein injection than via inhalation. For this experiment, we chose to compare fungal burden at the time of illness for the mice infected with the different strains. Specifically, organs from mice infected with the wild-type strain or the *CFT1* reconstituted strain were collected at day 7, and organs from mice infected with the *cft1* mutant were harvested at day 29. Similar to results from inhalation model, lower numbers of the *cft1* mutant cells were found in the brain, the spleen and the liver when the mice became ill at day 29 compared to infection with the wild-type strain at day 7 (Figure 10B). The result for the lung was a notable exception in that higher numbers of *cft1* mutant cells were found in mice infected with the *cft1* mutant at day 29 (Figure 10B), a phenomenon that was also observed in the inhalation model. We further confirmed the reduced ability of the *cft1* mutant to colonize the brain or lungs compared to the wild-type strain in a three-day time course after tail vein injection (Figure 10C). Overall, these data suggested that *CFT1* makes a contribution to virulence during cryptococcosis, and that transferrin or other iron sources dependent on Cft1 could potentially be primary sources for C. neoformans within the host, especially in the brain.

## Discussion

Iron uptake and homeostasis functions are known to be important for the pathogenesis of C. neoformans because iron overload exacerbates experimental cryptococcosis and iron levels influence the expression of virulence factors [26,36,43]. Pioneering physiological studies indicate that C. neoformans possesses at least two iron uptake systems [31,32,44]. The first is the utilization of siderophores although the fungus does not appear to produce these molecules [31]. The other is a reductive iron uptake system in which ferric iron is reduced to the ferrous form by cell surface reductase activity, by secreted reductants and by melanin in the cell wall [32,44]. Jacobson *et al.* (1998) detected activities of both high- and low-affinity iron uptake systems from cell cultures of C. neoformans, estimated the *K_m_* of the high-affinity uptake to be 0.6 μM and found that the activity of the high iron uptake system is down-regulated by 15 μM of iron [31]. The low-affinity uptake system has not been characterized. In the context of these findings, we determined the roles of the *CFT1* and *CFT2* genes that show sequence similarity to genes for the iron permeases responsible for high-affinity iron uptake in other fungi such as S. cerevisiae and C. albicans. Our analysis of mutants defective in *CFT1*, *CFT2* or both genes revealed that *CFT1* plays the major role in iron acquisition in C. neoformans. The *cft1* mutant displayed growth defects in the presence of iron sources (FeCl_3_ and transferrin) that are known to be transported by reductive iron uptake system in C. albicans [13]. These results, together with our expression data showing that *CFT1* is down-regulated at higher iron concentrations, suggest that Cft1 functions as a high-affinity iron permease responsible for iron uptake in C. neoformans. However, Cft1 is not involved in the heme or siderophore uptake systems in C. neoformans because we found that the *cft1* mutant displays normal growth with these iron sources.

The role of Cft2 in C. neoformans is less clear because of the low transcript level for the gene and the lack of robust phenotypes for the *cft2* mutant. Several lines of evidence suggest that Cft2 may play a minor role and may function redundantly with Cft1. First, the *cft1 cft2* double mutants displayed a growth defect in the rich medium YPD (an iron-replete condition), whereas the *cft1* and *cft2* single mutants did not show growth defects in this medium. Second, the expression of *CFT2* is up-regulated in the *cft1* mutants and in response to iron limitation. Finally, the *cft1 cft2* mutant showed attenuated virulence compared with the single *cft1* mutant. It is possible that *CFT2* encodes a low affinity iron permease or, alternatively, that it encodes a vacuolar permease that functions to transport stored iron to the cytoplasm. It is also possible that the expression of *CFT2* may be different (e.g., higher), and that the gene may make a contribution, under conditions not tested in our study. Further biochemical analyses and localization studies will be needed to better define the role of Cft2.

The presence of two candidate iron permease genes, *CFT1* and *CFT2*, in C. neoformans is, at first glance, similar to the situation in C. albicans where the iron permease genes *CaFTR1* and *CaFTR2* have been characterized [12]. As was found with *CaFTR1*, *CFT1* appears to play a major role in iron uptake, growth on specific iron sources and virulence. A mutant lacking *CaFTR2* is similar to the *cft2* mutant in that neither showed growth phenotypes and neither is required for virulence. However, the regulation of *CaFTR2* is quite different because it has a low transcript level in low iron conditions and an elevated transcript at higher levels. Ramanan and Wang [12] suggest that the differential expression of *CaFTR1* and *CaFTR2* may reflect their functions in different environments. Similarly, *CFT2* may be expressed under specific conditions and, in this context, it is interesting that Fan et al. [37] found that *CFT2* and *CFO2* transcript levels were induced upon phagocytosis. Thus, these genes may make a contribution during infection consistent with the observed additional attenuation in virulence found for the *cft1 cft2* mutant compared with the *cft1* mutant.

Previously, we described the role of the GATA type zinc finger transcription factor Cir1 in iron-related regulation in C. neoformans [26]. Microarray analysis revealed that Cir1 regulates expression of genes required for iron uptake in both a negative and a positive manner [26]. In that report, we found that a putative iron permease (XP_569788) is a negatively regulated, downstream target of Cir1 in the serotype D strain B3501A. The current study revealed that this gene is equivalent to *CFT2* in the serotype A strain H99. We also identified another iron permease gene (XP_568258) from the microarray experiments that we now know corresponds to *CFT1*. We should note that values representing differential expression of the *CFT1* ortholog (XP_568258) were not statistically significant in the microarray analysis in the serotype D strain background. The current study using a serotype A strain revealed that *CFT1* is a positively-regulated downstream target gene of Cir1, which is opposite to the regulation found for *CFT2*. This confirms our previous findings of a dual mode of Cir1 regulation in C. neoformans [26]. The *SIT1* gene, which encodes the siderophore transporter [33], also belongs in the regulatory network of genes positively controlled by Cir1.

As mentioned, the cAMP pathway negatively regulates genes involved in the high affinity iron uptake through the protein kinase A subunit Tpk2 in S. cerevisiae. U. maydis also possesses similar connections but the pathway positively regulates genes involved in the high affinity uptake [24]. In our study, we observed that the cAMP pathway regulates transcriptional levels of iron permease genes in C. neoformans. An ∼2-fold reduction in *CFT1* transcript levels was found in the *pkr1* mutant and a marked elevation of the *CFT2* transcript was found in the *pka1* mutant (∼10-fold). These data suggest that regulation of *CFT1* by the cAMP pathway may be indirect and of minor significance under the conditions we tested. Also, the *pka1* mutant did not show a reciprocal pattern of differential expression compared with the *pkr1* mutant in terms of *CFT1* transcript levels, again suggesting that the influence may be indirect. The more substantial regulation of *CFT2* by Pka1 further supports the idea that there may be specific conditions where Cft2 makes a contribution to iron acquisition or other functions. The reason why Pka1 controls *CFT2*, but not *CFT1*, remains to be investigated. Finally, the regulation by cAMP extends to other genes involved in iron acquisition in C. neoformans because we previously found elevated transcripts for the *SIT1* gene (siderophore transporter) in the *pka1* mutant [33].

The expression of the *CFT1* and *CFO1* genes was recently shown to be influenced by deletion of the regulatory gene *SRE1*, which functions in an oxygen sensing pathway in C. neoformans [45]. Based on predictions from work on Sre1 in S. pombe, Chang et al. [45] found that Sre1 in a serotype D strain of C. neoformans is activated by inhibition of sterol synthesis and low oxygen levels, and that the gene is required for wild-type levels of growth under hypoxic and low iron conditions. A microarray experiment to compare the transcriptomes of wild type and *sre1* mutant cells grown in a low oxygen environment revealed that Sre1 positively influences the expression of ∼100 genes. These genes encoded enzymes for ergosterol biosynthesis as well as iron transport functions such as Cft1 and Sit1 [33], and the copper transporter Ctr4 [46]. Deletion of *SRE1* negatively influenced the expression of another 414 genes and many of these encoded stress-related functions. The role of the *SRE1* gene has also been characterized in a serotype A strain of C. neoformans [47]. In this strain background, the *sre1* mutant displays a hypoxia-sensitive phenotype, slight defects in capsule and melanin formation, and a reduced ability to proliferate and cause disease in a mouse model of infection. Interesting, Chun et al. [47] did not observe the changes in transcript levels for the *CFT1* and *CFO1* genes as a result of hypoxia or loss of *SRE1* that were seen by Chang et al. [45]. This may reflect differences in the regulation of iron uptake functions between strains of the A and D serotypes.

The connections between oxygen, iron and sterol biosynthesis established by Chang et al. [45] are interesting in light of our finding that the *cft1* mutant displayed increased sensitivity to an inhibitor of ergosterol biosynthesis. This observation further supports the idea that C. neoformans, as a strict aerobe, must balance iron availability with oxygen levels and ergosterol synthesis [26,45]. Similar connections have been described in *S. cerevisiae,* although this fungus is capable of anaerobic growth. In yeast, anaerobiosis results in reduced heme synthesis, a lower rate of synthesis of respiratory proteins and loss of the ability to synthesize sterols because of the iron dependence of enzymes in the pathway [48].

*SRE1* in C. neoformans is also required for the establishment and growth of the fungus in the brains of infected mice thus indicating that this tissue site may be limited for oxygen, and perhaps for iron. Our finding that the *cft1* mutant has reduced colonization of the brain further suggests that this tissue is limited for iron. Additional evidence for integration of iron, oxygen and sterol biosynthesis comes from the finding that a 2.2-fold reduction was seen for *SRE1* transcript levels under the iron limited condition in the *cir1* mutant [26,45]. Thus, *SRE1* may be a direct or indirect target of Cir1 regulation and the two regulators could also potentially interact at the promoters of genes such as *CFT1*. Additionally, these factors both influence the transcript levels of the copper transporter Ctr4. Waterman et al. [46] showed that this gene is activated by the transcription factor Cuf1 and, in parallel with the findings for the *sre1* and *cft1* mutants, *cuf1* mutants show reduced CNS colonization in a murine model of infection. Overall, these results provide the first glimpses of the integration of oxygen, sterol, copper and iron sensing regulatory schemes that influence virulence and CNS colonization in C. neoformans.

Our analysis revealed that the *cft1* mutant (but not the *cft2* mutant) showed reduced virulence in the mouse model of the cryptococcosis, a result consistent with a role for Cft1 in iron acquisition in vivo. This is consistent with SAGE experiments on C. neoformans cells from a rabbit model of cryptococcal meningitis and from a mouse pulmonary infection which show that *CFT1* transcript was abundant during growth in the host [35] (Hu and Kronstad, manuscript in preparation). *CFT2* appears to make a minor contribution because the *cft1 cft2* double mutant showed a further reduction in virulence compared to the *cft1* mutant. Two models of cryptococcosis (inhalation and tail vein injection) revealed reduced numbers of fungal cells in the brains, spleens and livers of mice infected with the *cft1* mutant compared to mice infected with the wild-type and reconstituted strains. Overall, it appeared that the mutant exhibited a generalized growth defect that could account for the slower disease progression. In particular, we noted a delayed appearance of the fungal cells in brains of mice infected with the *cft1* mutants by either route. The *cft1* mutant cells did eventually reach high levels in the lungs near the time that the mice succumbed to infection. The higher burden was particularly striking compared with the low numbers in the brain at the same time. This observation may indicate that lung tissue provides a growth environment in which the requirement for Cft1 is not as stringent as in the brain. For example, tissue differences in oxygen availability may influence the requirement of the fungus for iron [49].

The delayed disease progression for the *cft1* and the *cft1 cft2* mutants supports our hypothesis that C. neoformans is partially dependent on an iron source that requires a reductive uptake system during infection. Transferrin is likely the source because of its abundance and because the *cft1* mutant showed defects in the utilization and uptake of iron from transferrin *in vitro*. We hypothesize that transferrin may be particularly important for the growth of C. neoformans in the brain. Transferrin may be the primary iron source in the CNS because it is the only iron carrier protein that can be transported through the blood-brain barrier (BBB) [50]. Although a reduction in fungal burden in mice infected with the *cft1* mutant was apparent, the mutant eventually caused mortality. We propose that the *cft1* mutant (and the *cft1 cft2* mutant) was able to grow in the host because it could also utilize iron sources such as heme via a non-reductive mechanism. This idea is consistent with our finding that wild-type cells showed robust growth in the presence of heme or siderophores. The mechanisms of heme utilization have not been investigated in C. neoformans.

C. neoformans must breach the BBB and invade the CNS to cause meningoencephalitis. Only a few pathogenic microbes are able to cross the BBB and the process is best understood in bacteria. For example, the bacterium Listeria monocytogenes infects leukocytes and is then transported into the CNS during leukocyte migration through the BBB (i.e., the “Trojan horse” mechanism) [51]. The encapsulated bacteria Streptococcus pneumoniae and *Neisseria. meningitis* use transcytosis to cross a monolayer of brain endothelial cells [52,53]. Evidence to date indicates that C. neoformans is also able to adhere to and transcytose across human brain microvascular endothelial cells [54]. In this context, we speculate that *CFT1* may play an iron acquisition role while the fungus is within the endothelial cell during transcytosis and/or while the fungus is within CNS. Interestingly, holo-transferrin also enters the endothelial cells of the BBB by transcytosis so this iron source would potentially be available to the fungus [55]. The mechanisms of iron acquisition by C. neoformans during phagocytosis by macrophages also remains to be investigated. As mentioned, *CFT2* transcripts are elevated upon phagocytosis but we note that recent studies show that phagosome extrusion of the fungal cells occurs as early as 2 hours after uptake [56,57]. Overall, our results provide insights into the role of iron acquisition functions in cryptococcal disease, reveal iron source preferences, and suggest possible targets for antifungal therapy, especially in the context of treating fungal meningitis.

## Materials and Methods

### Strains and growth conditions.

The strains used in this study (Table S1) were routinely grown in yeast extract, bacto-peptone medium with 2.0% glucose (YPD, Difco) or yeast nitrogen base (YNB, Difco) with 2.0% glucose. Defined low iron medium was prepared as described [58] and we have determined that this medium contains approximately 1.3 μM iron (data not shown). This medium was used for the experiments described in Figures 2–4, 6, and 7, and 0 μM in the figure labels indicates that no additional iron was added. Iron-replete medium was prepared by adding the iron sources FeCl_3_, holo-transferrin, heme or feroxamine into low-iron medium at the final concentrations indicated in the text. To assess growth, cells were first grown in low-iron medium for two days at 30°C to deplete intracellular iron stores and to fully induce the high affinity iron uptake system in *C. neoformans,* as suggested by Jacobson et al. [31]. The number of cells was determined using a haemocytometer and 2.0 × 10^4^ cells were transferred to the wells of a 96-well plate containing low-iron medium as a control or low-iron medium containing different iron sources. Iron sources were diluted by serial two-fold dilutions in a total volume of 200 μl. The plates were incubated at 30°C for three days, and the optical density of each well was read with a microtitre plate reader at 595 nm. Additional experiments to examine growth rate were performed in 5 ml cultures containing low iron medium supplemented with the same iron sources. For antifungal sensitivity tests on plates, 10-fold serial dilutions of cells were spotted onto YPD plates containing miconazole or amphotericin B. Plates were incubated at 30°C for two days.

### Construction of mutant strains.

The locus numbers for *CFT1* and *CFT2* in the C. neoformans serotype A genome are CNAG_06242.1 and CNAG_02959.1, respectively (http://www.broad.mit.edu/annotation/genome/cryptococcus_neoformans). The sequences for these genes were used for mutant construction. All primers used for the experiments are listed in Table S2. To construct a *cft1* mutant, the genomic region of 1740 bp that corresponds to the entire coding sequence of *CFT1* was replaced by a disruption cassette containing the nourseothricin acetyltransferase gene (*NAT*) using 5' and 3' flanking sequences of *CFT1*. The disruption cassette was constructed by an overlap PCR method using primers TL2061, TL2062, TL2063, TL2064, TL2065 and TL2066, along with strain H99 genomic DNA and the plasmid pCH233 as templates [59,60]. The construct was biolistically transformed into the wild-type strain as described previously [61]. Positive transformants were identified by PCR, confirmed by Southern blot analysis and named TLF1–9 (Figure S3A). To reconstitute the *cft1* mutant, primers H9F1RCF and H9F1RCR were used to amplify the wild type *CFT1* gene from genomic DNA. The PCR fragments were digested with SacI and SpeI, and cloned into pJAF to construct pWH045 containing the neomycin resistant marker (*NEO*). The plasmid pWH045 was digested with NdeI and transformed into the *cft1* mutant. Positive transformants containing the wild-type *CFT1* at its authentic locus were identified by PCR and named CFT1R9–4. To construct the *cft2* mutant, the genomic region of 2010 bp that corresponds to the entire coding sequence of *CFT2* was replaced by a disruption cassette containing the *NEO* marker using 5' and 3' flanking sequences of *CFT2*. Primers TL2071, TL2072, TL2073, TL2074, TL2075 and TL2076 were used for construction of the disruption cassette by an overlap PCR method and H99 genomic DNA and the plasmid pJAF were used as templates [59,60]. The construct was transformed as described above and positive transformants were identified by PCR, confirmed by Southern blot analysis and named TLF2–9 (Figure S3B). To reconstitute the *cft2* mutant, primers H9F2RCFBglII and H9F2RCRBglII were used to amplify the wild type *CFT2* gene. The PCR fragments were digested with BglII and cloned into BamHI digested pCH233 to construct pWH056 containing the *NAT* marker. The plasmid pWH056 was digested with NdeI and transformed into the *cft2* mutant. Positive transformants containing the wild-type *CFT2* at its authentic locus were identified by PCR and named CFT2R9–2. The *cft1 cft2* double mutants were constructed by transforming the *CFT2* disruption cassette into the *cft1* mutant. The positive transformants were selected by PCR, confirmed by Southern blot analysis and named *cft1Δ cft2Δ* (Figure S3C).

### Iron uptake assays.

Iron uptake assays were performed as described previously with minor modifications [62,63]. Briefly, cells were grown in YNB medium overnight, transferred to YNB medium containing 1 mM ascorbic acid and 1mM ferrozine and incubated at 30°C for another 12 h. For iron uptake from FeCl_3_, 2.0 × 10^7^ cells were withdrawn from each culture, centrifuged and washed once with uptake buffer (0.2 M 3-(N-Morpholino)-propanesulfonic acid (MOPS), 2% glucose, 20 mM Na-citrate pH 6.8). Cells were resuspended in 0.5 ml of uptake buffer and kept at room temperature for 15 min for equilibration. Uptake buffer (0.5 ml) containing 20 μM of ^55^FeCl_3_was added to each sample, and the cells were incubated for 30 min at room temperature. After incubation, 5 ml of quenching buffer (0.375 M Succinic acid, 0.625 M Tris, 0.128 M EDTA, pH 6.0) was added, and each sample was immediately vacuum-aspirated through a GF/A filter (Whatman). The filter was washed with 20 ml of quenching buffer, and radioactivity was measured by liquid scintillation counting.

The uptake of iron from transferrin was performed as described previously [13]. Briefly, human apo-transferrin was purchased from Sigma and ^55^Fe labelled transferrin was prepared by the addition of a threefold molar excess of ^55^FeCl3 to 10 μM apo-transferrin in transferrin loading buffer (0.1 M HEPES, pH 7.5, 0.15 M sodium chloride, and 10 mM sodium bicarbonate). The reaction mix was incubated for 30 min at 22°C and 30 min on ice, and the labelled transferrin was separated from unbound ^55^Fe using Sephadex G-25 spin columns. Cells were grown to mid log phase in the presence of 300 μM Ferrozine. They were then washed three times with citrate-glucose buffer (0.1 M Morpholinethanesulfonic acid (MES) buffer pH 6.0, 20 mM Na-citrate, 2% glucose) and resuspended in the same buffer followed by incubation in a 30°C water bath for 15 min prior to the addition of 2 μM ^55^Fe-transferrin. After 1 h, samples were removed and quenched using 3 ml of quenching buffer. The free iron was removed by washing and the ^55^Fe radioactivity associated with the cells was measured by liquid scintillation counting on Wallac 1409 liquid scintillation counter.

### Quantitative real-time RT-PCR.

Primers for real-time RT-PCR analysis were designed using Primer Express software 3.0 (Applied Biosystems) and are listed in Table S3. Cell cultures were prepared by growth in low-iron medium as described above, and then transferred to the same medium containing different concentrations of iron sources as indicated in the text. Total RNA was purified with the RNeasy kit (Qiagen), treated with DNAse (Qiagen) and cDNA was generated using the SuperScript First-Strand Synthesis System (Invitrogen). PCR reactions were monitored as described previously [33], and relative gene expression was quantified using the SDS software 1.3.1 (Applied Biosystems) based on the 2^-ΔΔ*CT*^ method [64]. *ACT1* was used as a control for normalization.

### Virulence assays.

To examine virulence in an inhalation model of cryptococcosis, strains were cultured in 5 ml YPD overnight at 30°C. The overnight cultures were washed twice with PBS, and the fungal cells were resuspended in PBS. 4–6 week old female A/Jcr mice were anesthetized intraperitoneally with ketamine (80 mg/kg) and xylazine (5.5 mg/kg) in saline, and suspended on a silk thread by the superior incisors. A cell suspension of 5 × 10^4^ cells in 50 μl was slowly dripped into the nares of the anesthetized mice, and the mice were suspended for 10 min on the thread. For the tail vein injection model, strains were directly injected in the lateral tail veins of mice at a density of 5 × 10^4^ cells in 200 μl of PBS. The status of the mice was monitored daily post-inoculation. Mice reaching the humane endpoint were euthanized by CO_2_ anoxia. To assess fungal burden of organs from mice infected by inhalation or tail vein injection, three mice were used for each strain at each time point. Mice were euthanized with CO_2_, four organs including the brain, the lungs, the liver and the spleen, were aseptically removed and soaked in 0.5 ml PBS overnight at 4°C. Organs were homogenized manually with a sterile plastic pestle. Cell strainers (BD, 70 μm Nylon) were used to remove tissue debris in the samples. The samples were serially diluted and 200 μl of the diluted samples were plated and spread with sterile 4 mm glass beads on YPD plates containing chloramphenicol (35 μg/ml). CFUs were determined after three days of incubation at 30°C.

The following method was used to examine the timing of dissemination to the brain and lungs following tail vein injection. Cells were cultured in 5 ml of YPD overnight at 30°C, washed twice with PBS, and resuspended in PBS. Cell counts were performed with a haemocytometer. A total of 28 6-week-old female A/Jcr mice were inoculated with 200 μl of the wild-type strain H99 or the *cft1* mutant (2.5 × 10^5^ cells/ml in PBS). The brain and the lungs of the mice were harvested aseptically on days 1, 2 and 3 post-inoculation. The organs were manually homogenized in 1 ml PBS with sterile plastic pestles. Cell strainers (BD, 70 μm nylon) were used to remove tissue debris in the samples. The samples were serially diluted and 200 μl of the dilutions were plated followed by incubation at room temperature for 3 days.

The protocols for the virulence assays (protocol A99–0252) conformed to regulatory standards of and was approved by the University of British Columbia Committee on Animal Care.

## Supporting Information

Figure S1 Growth of Strains in YPD Medium (27 KB TIF)

Figure S2 Analysis of the Rate of Growth for the Mutant Strains in Low-Iron Medium with and without FeCl_3_ or Heme as the Iron Source (194 KB TIF)

Figure S3 Disruption of Wild-Type *CFT1* or *CFT2* Was Confirmed by Southern Blot Analysis (360 KB TIF)

Table S1 Strains Used in This Study (28 KB DOC)

Table S2 Primers Used for Construction of Mutant Strains (20 KB DOC)

Table S3 Primers Used for Real-Time RT-PCR (20 KB DOC)
