# Supplementary material for: Iron Source Preference and Regulation of Iron Uptake in Cryptococcus neoformans
Source: PLoS Pathog. 2008 Feb 15;4(2):e45. doi: 10.1371/journal.ppat.0040045 (PMC2242830; doi:10.1371/journal.ppat.0040045)
Supplement: Supplementary file 1 [file ppat.0040045.st001.doc]

| Strains | Genotype | References |
| --- | --- | --- |
| H99 | *MATα* | [1] |
| TLF1-9 | *MATα cft1Δ ::NAT* | This study |
| CFT1R9-4 | *MATα cft1Δ ::NAT, CFT1-NEO* | This study |
| TLF2-9 | *MATα cft2Δ ::NEO* | This study |
| CFT2R9-2 | *MATα cft2Δ ::NEO, CFT2-NAT* | This study |
| cft1cft2 | *MATα cft1Δ ::NAT, cft2Δ ::NEO* | This study |
| CDC1 | *MATα pka1Δ ::ADE2 ade2* | [2] |
| JKH4 | MATα pka2Δ ::URA5 ura5 | [3] |
| CDC7 | *MATα pkr1Δ ::URA5 ura5* | [2] |

**Table S1. Strains used in this study.**

1. Perfect JR, Ketabchi N, Cox GM, Ingram CW, Beiser CL (1993) Karyotyping of *Cryptococcus neoformans* as an epidemiological tool. J Clin Microbiol 31: 3305-3309.

2. D'Souza CA, Alspaugh JA, Yue C, Harashima T, Cox GM, et al. (2001) Cyclic AMP-dependent protein kinase controls virulence of the fungal pathogen *Cryptococcus neoformans*. Mol Cell Biol 21: 3179-3191.

3. Hicks JK, D'Souza CA, Cox GM, Heitman J (2004) Cyclic AMP-dependent protein kinase catalytic subunits have divergent roles in virulence factor production in two varieties of the fungal pathogen *Cryptococcus neoformans*. Eukaryot Cell 3: 14-26.
