# Supplementary material for: Iron Source Preference and Regulation of Iron Uptake in Cryptococcus neoformans
Source: PLoS Pathog. 2008 Feb 15;4(2):e45. doi: 10.1371/journal.ppat.0040045 (PMC2242830; doi:10.1371/journal.ppat.0040045)
Supplement: Supplementary file 2 [file ppat.0040045.st002.doc]

| Name | Sequence |
| --- | --- |
| TL2061 | GTGGTTGAAGAACATGCCGTGAGAA |
| TL2062 | AATTCCAGCACACTGGCGGCCGTTACTAGTGTCTGTCTTGGGTCAGGGATGTTAC |
| TL2063 | AATTCTGCAGATATCCATCACACTGGCGGCCTGAAGGCAAGAGTCCGGTACTGAC |
| TL2064 | AACTACAGTTTGTCGTCGCATCAGA |
| TL2065 | GAGAATGAAGGGATGTACCGACGG |
| TL2066 | GGCCATTGTCTTTAGTACGGAGGT |
| H9F1RCF | AAGGGTGAGCTCCTGCATCCCAGTTGTATACTGTAAGCTGAAC |
| H9F1RCR | AGTTTGACTAGTAAACTCCACTGTATATTGCTGGGGC |
| TL2071 | CTTCTTGTTGTTGACCTCGGGCATT |
| TL2072 | AATTCCAGCACACTGGCGGCCGTTACTAGTGCCGCTTCGACCGTCTCAC |
| TL2073 | AATTCTGCAGATATCCATCACACTGGCGGCTAATTTGGGAGTCATGCTACATTG |
| TL2074 | GGCGCGGTCATTCTGGATACTTT |
| TL2075 | GGCATTCTCTCGCAAAGG |
| TL2076 | CCGATTACCGTACATGGATCTC |
| H9F2RCFBglII | CTCAATCAGATCTCTCAGTGTTCGGGTCTAGCAATCGAC |
| H9F2RCRBglII | AAGCAAGAGATCTAAGAAGAGAGATATTGGCGGTGGCC |

**Table S2. Primers used for construction of mutant strains.**
