# Supplementary material for: Iron Source Preference and Regulation of Iron Uptake in Cryptococcus neoformans
Source: PLoS Pathog. 2008 Feb 15;4(2):e45. doi: 10.1371/journal.ppat.0040045 (PMC2242830; doi:10.1371/journal.ppat.0040045)
Supplement: Supplementary file 3 [file ppat.0040045.st003.doc]

**Table S3. Primers used for real-time RT-PCR**

| Primers | | Sequence |
| --- | --- | --- |
| CFT1 | Forward | ACGAGAACGACCTCAGTAATGAAA |
|  | Reverse | CTGCGTCCCCAGCTGATC |
| *CFT2* | Forward | CCGGCAAGGCCCTCTT |
|  | Reverse | TGTCAGGCTCGCCATCTTC |
| *SIT1* | Forward | TGAGGACTACCCGAGACTTGGT |
|  | Reverse | TTTCGAGCTTCTCGGCATCT |
| *ACT1* | Forward | CCACACTGTCCCCATTTACGA |
|  | Reverse | CAGCAAGATCGATACGGAGGAT |
